# Abnormal left ventricular global longitudinal strain by speckle tracking echocardiography in COVID-19 patients

**DOI:** 10.2217/fca-2020-0121

**Published:** 2020-10-09

**Authors:** Lori B Croft, Parasuram Krishnamoorthy, Richard Ro, Malcolm Anastasius, Wenli Zhao, Samantha Buckley, Martin Goldman, Edgar Argulian, Samin K Sharma, Annapoorna Kini, Stamatios Lerakis

**Affiliations:** ^1^The Zena & Michael A. Wiener Cardiovascular Institute, Icahn School of Medicine at Mount Sinai, NY 10029, USA

**Keywords:** coronavirus, COVID-19 infection, myocardial strain imaging, speckle tracking echocardiography, transthoracic echocardiography

## Abstract

COVID-19 infection can affect the cardiovascular system. We sought to determine if left ventricular global longitudinal strain (LVGLS) is affected by COVID-19 and if this has prognostic implications. **Materials & methods:** Retrospective study, with LVGLS was measured in 58 COVID-19 patients. Patients discharged were compared with those who died. **Results:** The mean LV ejection fraction (LVEF) and LVGLS for the cohort was 52.1 and -12.9 ± 4.0%, respectively. Among 30 patients with preserved LVEF(>50%), LVGLS was -15.7 ± 2.8%, which is lower than the reference mean LVGLS for a normal, healthy population. There was no significant difference in LVGLS or LVEF when comparing patients who survived to discharge or died. **Conclusion:** LVGLS was reduced in COVID-19 patients, although not significantly lower in those who died compared with survivors.

## Lay abstract

COVID-19 infection can affect the cardiovascular system including the heart muscle. Myocardial strain measures the function of the heart muscle at cell level and can pick up unusual heart function before routine measurements. Ejection fraction (how hard the heart muscle is squeezing) can be seen with our own eyes, but strain cannot and needs special techniques to see it. In this study we wanted to find out what was the strain on COVID-19 patients and if there was a relationship between strain and the outcome of the patients (if they were able to recover from COVID-19). The important finding of our study is that strain was low in all patients with COVID-19 infection regardless of routine measurements when compared with patients without the infection. However, in this study we found no correlation between the low strain and the result of the infection for the COVID-19 patients.

COVID-19 infection, which is caused by the SARS-CoV-2, emerged at the end of 2019 in Wuhan, China. It was first described as a pneumonia-like illness, with the most common symptoms reported being fever, cough, fatigue and gastrointestinal distress [1]. In addition to causing a respiratory illness, SARS-COV-2 can have effects on multiple organ systems, including the cardiovascular system [2]. The mechanisms of cardiac injury include direct viral injury, oxygen supply/demand mismatch, hyperinflammatory state, epicardial coronary plaque rupture and stress cardiomyopathy [2]. Transthoracic echocardiography (TTE) is the initial imaging modality for evaluation of COVID-19 cardiac manifestations, and is useful in guiding clinical management [3].

Left ventricular global longitudinal strain (LVGLS) measured by speckle tracking echocardiography (STE), which tracks the displacement of speckles within the myocardium, provides an objective quantification of myocardial deformation with angle independence. It has been used in clinical practice for its diagnostic and prognostic data independent of LV ejection fraction (LVEF) in patients with LV hypertrophy, in cardio-oncology, valvular diseases and both ischemic and nonischemic cardiomyopathies [4]. Furthermore, right ventricular free-wall strain was recently shown to have prognostic implications in COVID-19 infected patients [5]. In this retrospective study, we sought to determine if LVGLS is affected by COVID-19 infection and if it has implications on clinical outcomes.

## Materials & methods

### Study population

The research protocol was approved by the Institutional Review Board. This was a retrospective study of nonconsecutive patients hospitalized with COVID-19. Patients aged >18 years admitted to Mount Sinai Hospital, NY, USA, and tested positive for SARS-CoV-2 using reverse transcriptase PCR assay from nasopharyngeal swabs and who subsequently underwent TTE during the hospitalization were included.

### Patient data collection

Demographic data were collected for all patients. Baseline laboratory values of significance in patients diagnosed with COVID-19 were obtained, including hemoglobin, full blood count and differential (white blood cell count), estimated glomerular filtration rare. Inflammatory markers such as C-reactive protein (CRP), D-dimer and troponin were also collected [6].

### Transthoracic echocardiography

Patients underwent TTE using the EPIQ echocardiography system (Philips North America, MA, USA). TTE studies were performed using a focused examination protocol according to American Society of Echocardiography recommendations for use of echocardiography in patients with confirmed COVID-19 infection [3]. All studies were reviewed by two expert echocardiographers (S Lerakis and LB Croft) and only patients with optimal apical 3-, 2- and 4-chamber views were included into the study. Measurements of LVGLS were performed offline using QLab 13.0 (Philips, Best, The Netherlands).

### Clinical study outcomes & statistical analysis

Patients discharged from hospital were compared with those who died or required mechanical ventilation. Categorical variables were presented as percentage (%), and continuous variables as mean ± standard deviation for normally distributed variables and median interquartile range (IQR) for others. Shapiro–Wilk test and histogram were used to test normality for each variable. Student’s *t*-test was performed for normally distributed continuous variables and Mann–Whitney *U* test for nonparametric continuous variables. Chi-square test was performed for categorical variables to examine if there were significant differences between the groups. Kaplan–Meier survival analysis and Cox regression was performed to determine the association between LVGLS and the combined outcome of death or need for mechanical ventilation, by comparing patients greater and less than an arbitrary 75th centile LVGLS threshold, with the follow-up period as time since echocardiogram. Variables selected for entry into the model were those with a p < 0.1 on Cox univariate analysis. The Cox regression modeling considered all patient demographics, inflammatory biomarkers and LVEF. Statistical analysis was performed using STATA 14.0 MP (StataCorp LP, TX, USA).

## Results

**Figure 1.**
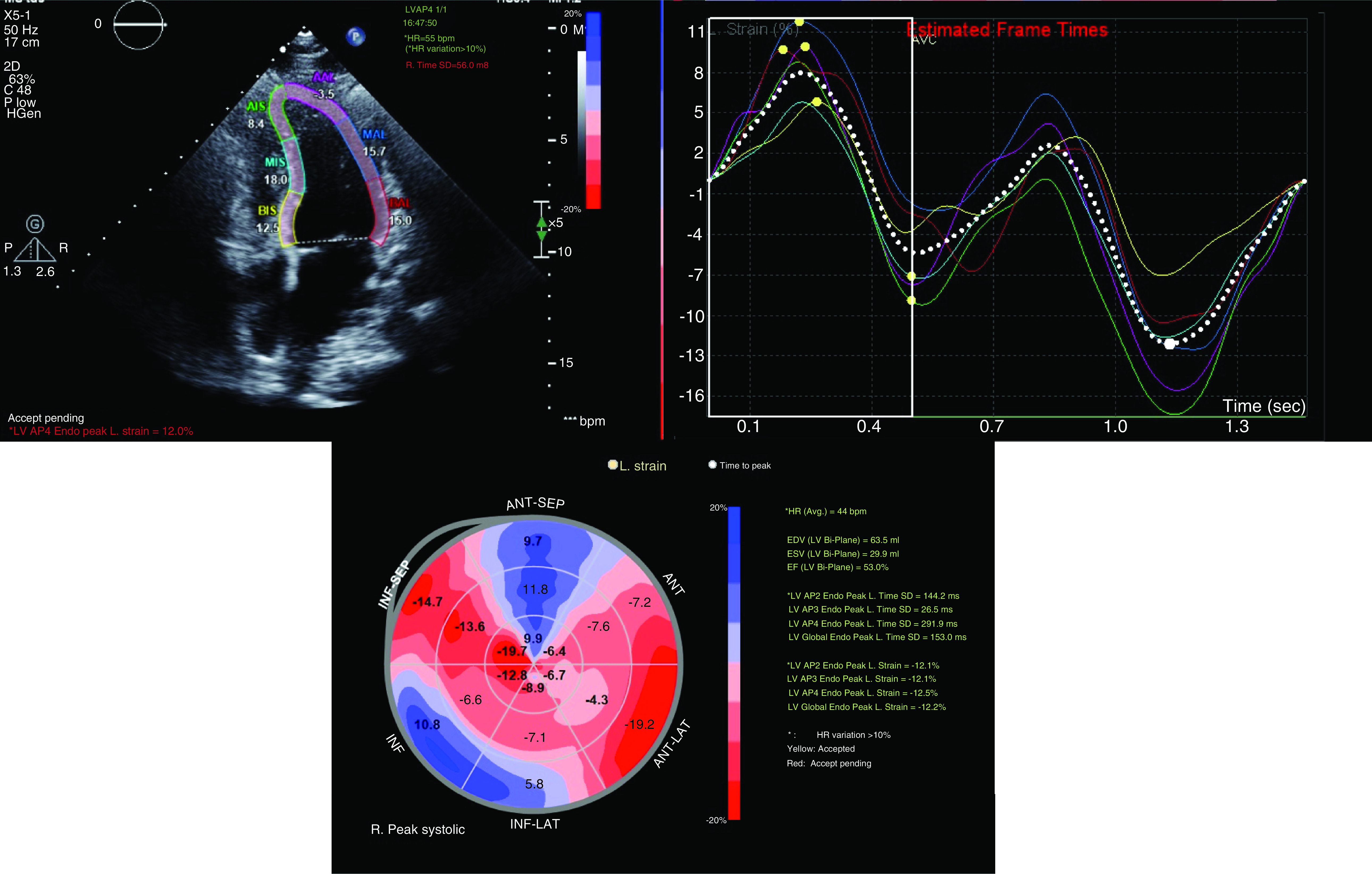
Example of left ventricular global longitudinal analysis in a patient with COVID-19 infection. Patient had a preserved LVEF (>50%), and 2D speckle tracking analysis demonstrated a LVGLS of -12.0%. LVEF: Left ventricular ejection fraction; LVGLS: Left ventricular global longitudinal strain.

From 103 COVID-19 positive patients who had a TTE performed in our institution, 58 (56.3%) had image quality suitable for LVGLS evaluation. An example of LVGLS analysis using 2D STE is shown in Figure 1. The mean age of the 58 patients was 54.1 ± 14 years and 58.6% were males (Table 1). Nine of the 58 patients died due to COVID-19 infection. The mean LVEF was 52.1%, with 30 patients (51.7%) having a LVEF >50% and the remainder (28; 48.3%) had an LVEF ≤50%. The mean LVGLS for the entire cohort was -12.9 ± 4.0%, and among the cohort of 30 patients with preserved LVEF it was -15.7 ± 2.8%. For comparison, the published reference data of mean LVGLS in normal, healthy patients with preserved LV function is -19.7% (95% CI: -20.4 to -18.9%) [7].

**Table 1.** Baseline patient demographics, laboratory and echocardiographic findings and in-hospital treatment.

Variables	Total (n = 58)	No death (n = 49)	Death (n = 9)	p-value^†^
Age (years)	54.1 ± 14	53.5 ± 14	57.7 ± 16	0.31
Males, n (%)	34 (58.6)	31 (63.3)	3 (33.3)	0.09
DM, n (%)	23 (39.7)	18 (36.7)	5 (55.6)	0.28
Hypertension, n (%)	40 (68.9)	33 (67.4)	7 (77.8)	0.53
BMI	26.9 (23.9, 29.8)	27.1 (24.1, 29.6)	26.9 (22.3, 30.1)	0.93
Prior CAD, n (%)	13 (22.4)	12 (24.5)	1 (11.1)	0.38
Afib/Aflutter, n (%)	7 (12.1)	6 (12.2)	1 (11.1)	0.92
HFrEF, n (%)	10 (17.2)	8 (16.3)	2 (22.2)	0.67
Asthma/COPD, n (%)	8 (13.8)	7 (14.3)	1 (11.1)	0.80
CRP	65.6 (28.4–119.6)	41.7 (24.6–104.5)	116.7 (85.9–159)	0.04
D-dimer	2.1 (1.1–3.7)	1.6 (1–3.5)	3.5 (2.6–12.6)	0.005
Troponin-I	0.04 (0.01–0.2)	0.03 (0.01–0.13)	0.06 (0.01–0.5)	0.65
WBC	8.2 (5.5–12.1)	8.5 (5.6–11.7)	6.3 (4.6–13.3)	0.43
Hemoglobin	12.2 ± 2.5	12.3 ± 2.4	11.4 ± 3.5	0.31
Platelet	244 ± 131	259 ± 132	162 ± 97	0.6
GFR	60 (28–60)	60 (27–60)	60 (30–60)	0.90
LVEF	52.1 (36–60.7)	53 (36–60.7)	44.7 (38.4–58)	0.60
GLS	12.9 ± 4	13.1 ± 4.4	11.8 ± 4.2	0.41
**Treatment**				
Hydroxychloroquine	38 (65.5)	32 (65.3)	6 (66.7)	0.9
Azithromycin	29 (50.0)	23 (39.7)	6 (66.7)	0.3
Corticosteroid	25 (43.1)	17 (34.7)	8 (88.9)	0.003
Convalescent Serum	3 (5.2)	2 (4.1)	1 (11.1)	0.4
Remdesivir	4 (6.9)	2 (4.1)	2 (22.2)	0.048
Anticoagulation	51 (87.9)	45 (91.8)	6 (66.7)	0.03
– Enoxaparin	24 (41.4)	20 (40.8)	4 (44.4)	
– Heparin	8 (13.8)	6 (12.2)	2 (22.2)	
– Apixaban	17 (29.3)	17 (34.7)	0	
– Rivaroxaban	2 (3.4)	2 (4.1)	0	

† Student’s *t* -test for normally distributed continuous variables and Mann–Whitney *U* test for nonparametric continuous variables. Chi-square test for categorical variables.

CAD: Coronary artery disease; COPD: Chronic obstructive pulmonary disease; CRP: C-reactive protein; DM: Diabetes mellitus; GFR: Glomerular filtration rate; GLS: Global longitudinal strain; HFrEF: Heart failure with reduced ejection fraction; LVEF: Left ventricular ejection fraction; LVGLS: Left ventricular GLS; WBC: White blood cell count.

**Figure 2.**
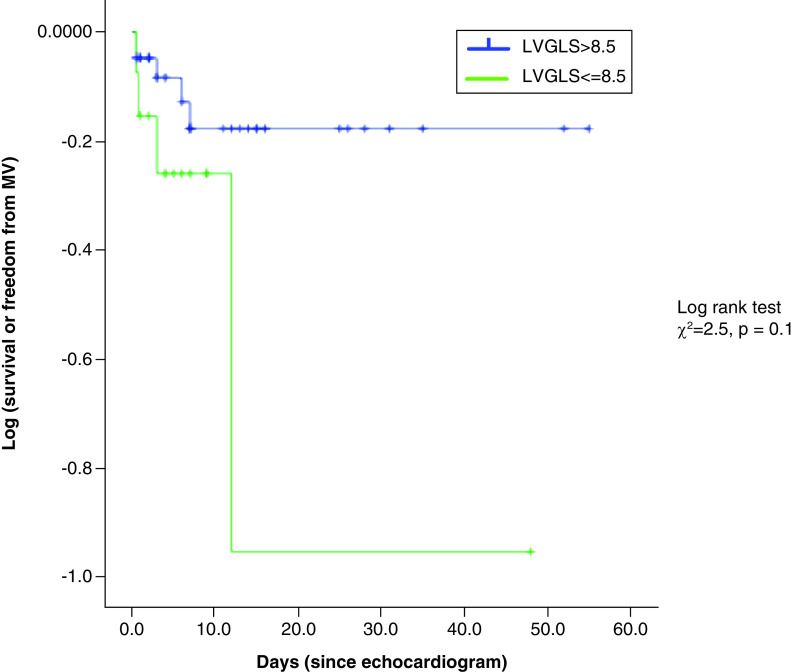
Kaplan–Meier survival curve depicting the prognosis associated with more severe reduction in left ventricular global longitudinal strain (global longitudinal strain ≤8.5). LVGLS: Left ventricular global longitudinal strain.

Patients who were discharged from hospital, as compared with those who died of COVID-19 infection, had lower CRP (41.7 vs 116.7, p = 0.04), and D-dimer levels (1.6 vs 3.5, p = 0.005). There was no significant difference in LVEF (53.0 vs 44.7%, p = 0.6) and LVGLS (-13.1 ± 4.4 vs -11.8 ± 4.2%, p = 0.41) when comparing patients who survived to discharge and those who died (Table 1). The administered pharmacological treatment was similar between these two patient groups, except corticosteroids were more commonly given to those who died (17.0 vs 88.9%, p = 0.003), and anticoagulation to those who survived to discharge (91.8 vs 66.7%, p = 0.03) (Table 1). Eleven patients following TTE died or required mechanical ventilation (MV). The study cohort was divided according to those with the most severe LVGLS reduction (75th centile) (>8.5 vs ≤8.5) and Cox regression analysis following consideration of patient demographics, laboratory parameters and LVEF, showed that there was a trend toward reduced in-hospital survival or need for MV among those with a LVGLS ≤8.5 (Figure 2). There were no significant independent predictors of death or need for mechanical ventilation.

## Discussion

Our study provides additional understanding of LVGLS changes in patients with confirmed COVID-19 infection. We observed that mean LVGLS of COVID-19 patients in this study cohort was lower than the reference mean LVGLS of a normal, healthy population. This was also true for those with preserved LVEF, suggesting the presence of occult myocardial injury. TTE may be the only practical imaging tool in the acute setting for detection of this injury, even when LVEF is preserved. Despite this decrease, LVGLS was not significantly different between patients who died and those who survived to discharge. More severe LVGLS reduction showed only a trend toward predicting in hospital death or need for mechanical ventilation. Inflammatory markers such as CRP and D-dimer at admission were significantly higher in patients who died compared with survivors.

While the LVGLS in COVID-19 infection was analyzed in the present study, recent studies have demonstrated right ventricular longitudinal strain to be a powerful predictor of mortality in patients with COVID-19 [5]. Different to our study, a prior study demonstrated a reduction in LVGLS in COVID-19 patients predicted mortality, independent of age and LVEF; the median LVEF in this cohort was 57.5% (interquartile range [IQ] 47.5–60) [8]. While it is difficult to completely reconcile the discrepancy in study findings, it is possible that the COVID-19 illness severity differed between the two study populations. Also, in the earlier study, Cox regression modeling adjusted for only age and LVEF [8], and it is uncertain if other patient characteristics, laboratory parameters or illness severity were considered.

The reduction in LVGLS with COVID-19 infection may be due to multiple factors. Myocardial injury may result through direct and indirect mechanisms. Direct mechanism is through viral infiltration of myocardium leading to cardiomyocyte death and inflammation. Indirect processes involve cardiac stress resulting from insults such as respiratory failure and hypoxemia, and cardiac inflammation in the setting of profound systemic hyperinflammation [2,9]. Principal components of the myocardial injury in COVID-19 are inflammatory mechanisms and activation of the immune response in the setting of underlying atherosclerosis, heart failure and hypertension [9]. In fact, nearly 70% of our study cohort had a history of hypertension. The study subjects who died had a significantly raised CRP, In keeping with the powerful inflammatory response in those with adverse COVID-19 infection outcomes. Prior retrospective studies have shown that nonsurvivors of COVID-19 infection had higher levels of CRP [10]. Acute cardiac injury and myocarditis may be caused by SARS-CoV-2 induced myocardial injury mediated by upregulation of the angiotensin-converting enzyme 2 receptor in the heart and coronary vessels [11,12]. Myocardial damage may also result from respiratory failure and hypoxia [11,12]. Immune mechanisms of myocardial inflammation may occur through activation of the innate immune response with liberation of pro-inflammatory cytokines and molecular mimicry leading to stimulation of the adaptive immune response [11–13]. In the setting of a more severe COVID-19 illness, these pro-inflammatory cytokines IL-6, IL-2 and TNF-α are released [14] resulting in a cytokine release syndrome [15], and trigger myocardial inflammation. Other mechanisms for myocardial injury include microvascular dysfunction, coronary plaque rupture and thrombosis. The majority of our study cohort had a preserved LVEF and in this subgroup, the LVGLS was reduced. Prior studies of patients with COVID-19 infection who also underwent comprehensive cardiac evaluation within 24 h of hospital admission showed that the LVEF was reduced in only a minority (10%) and those with an elevated troponin or more severe illness had an LVEF similar to patients with a nonraised troponin and a milder illness [16]. Furthermore, another study showed that LVEF was preserved and not significantly different between patients with COVID-19 infection who died and survived [5]. The use of STE and GLS is important in identification of the patient cohort with COVID-19 infection, preserved LVEF and occult myocardial injury marked by a reduction in LVGLS. It is known that lower LVGLS in large patient populations predicts future cardiac events including heart failure independent of LVEF, age, gender and hypertension [17]. The 2D GLS can predict all-cause mortality, with the incremental predictive power of GLS greatest in patients with normal or mildly reduced LVEF [18]. Thus, these patients with preserved LVEF and reduced LVGLS may benefit from closer long-term observation once recovered from the infection, for monitoring of long-term outcomes such as heart failure, arrhythmia or LV dysfunction.

Given the risk of COVID-19 infection transmission, the routine use of echocardiography is not encouraged. However, in the setting of clinical deterioration, TTE to evaluate for cardiac pathology in patients with COVID-19 infection should involve focused examinations to limit exposure time to the sonographer or physician performing the study [19]. Provided the focused examination includes appropriate apical 4-, 2- and 3-chamber views, then strain analysis can be performed using postprocessing software. Point-of-care ultrasound (POCUS) may be useful in the initial assessment for cardiac disease, while limiting staff exposure to COVID-19 infection [19]; however, this imaging modality may have limitations with image storage, and utility in postprocessing and performance of strain analysis. In particular right ventricle (RV) and LV strain decrease is recognized in COVID-19 [20], and POCUS cannot be used to detect these changes in myocardial strain. Thus, the presence of occult myocardial injury may be missed with the use of POCUS alone in patients with COVID-19 infection.

There are several limitations of the present study that should be addressed. This was a comparatively smaller study from a single institution in the epicenter of the pandemic in New York City. In particular the Cox regression analysis, showing a trend in the ability of LVGLS to predict need for mechanical ventilation or in-hospital death, was limited by a small study sample size. There are limitations to the generalizability of the findings, as most studies were limited in nature for safety reasons (to limit exposure to our sonographers), only 56.3% of the echocardiograms had adequate images for measurement of LVGLS. Many of these patients had difficult apical windows due to significant COVID-19 lung disease, limited patient positioning, body habitus and high positive end-expiratory pressures with mechanical ventilation. There may also have been selection bias given LVGLS was measured only in patients with adequate windows. For example, obesity has been linked with severe infections, which may also impact TTE image quality [21]. Given the retrospective study design, we cannot exclude the possible effects of confounding factors across the patient cohort, such as the presence of pre-existing LVGLS changes unrelated to COVID-19. Given this study did not have an analysis comparing COVID-19 patients to a matched control cohort with no COVID-19 infection, the reported low LVGLS values in the study cohort, may reflect changes due to factors unrelated to COVID-19. Thus, in our study cohort, direct cardiac injury due to COVID-19 infection cannot be firmly established. Also, measures of illness severity such as hemodynamic findings at the time of hospital admission with COVID-19 were not available for analysis.

## Clinical implications

LVGLS is reduced in patients hospitalized with COVID-19 infection, including among the majority of the study cohort with a preserved LVEF, indicating the presence of occult myocardial injury. Patients identified to have a reduction in LVGLS and preserved LVEF may need to be observed closely once recovered from the acute COVID-19 infection for future development of conditions such as heart failure, LV dysfunction or arrhythmia.

## Conclusion

The decrease in LVGLS among COVID-19 patients raises the possibility of the SARS-CoV-2 virus effects on LV myocardial function, which may be of concern, but does not provide definite prognostic information in terms of mortality or need for mechanical ventilation. Further studies with larger patient sample size, comparison to matched patient cohorts without COVID-19 infection and long-term follow-up are needed to understand the complete ramifications of the reduced LVGLS, including the relationship to future cardiac events.

**Summary points**

- SARS-COV-2, the cause of COVID-19, can have effects on multiple organ systems, including the cardiovascular system. The mechanisms of myocardial injury include direct viral injury, oxygen supply/demand mismatch, hyperinflammatory state, epicardial coronary plaque rupture and stress cardiomyopathy.
- Left ventricular global longitudinal strain (LVGLS) measured by speckle tracking echocardiography, provides an objective quantification of myocardial deformation with angle independence and can be utilized to detect myocardial injury.
- LVGLS is reduced in patients hospitalized with COVID-19 infection, including among the majority of the study cohort with a preserved LV ejection fraction (LVEF).
- The reduction in LVGLS in those with a preserved LVEF indicates the presence of occult myocardial injury.
- More severe LVGLS reduction showed only a trend toward predicting in hospital death or need for mechanical ventilation.
- Patients identified to have a reduction in LVGLS and preserved LVEF may need to be observed closely once recovered from the acute COVID-19 infection for future development of conditions such as heart failure, LV dysfunction or arrhythmia.
